# Creating sustainable capacity for river science in the Congo basin through the CRuHM project

**DOI:** 10.1098/rsfs.2023.0079

**Published:** 2024-08-09

**Authors:** Paul D. Bates, Raphaël M. Tshimanga, Mark A. Trigg, Andy Carr, C. A. Mushi, Pierre M. Kabuya, Gode Bola, Jeff Neal, Preksedis Ndomba, Felix Mtalo, Denis Hughes

**Affiliations:** ^1^ School of Geographical Sciences, University of Bristol, Bristol BS8 1SS, UK; ^2^ Congo Basin Water Resources Research and Capacity Building Center – CRREBaC, University of Kinshasa, DRC - P.O BOX: 1031, Kinshasa, Democratic Republic of Congo; ^3^ School of Civil Engineering, University of Leeds, Leeds LS2 9JT, UK; ^4^ School of Civil & Environmental Engineering, Ardhi University, Dar es Salaam, Tanzania; ^5^ Congo Hydrological Research Unit (UReHC), Department of Natural Resources Management, Faculty of Agronomic Sciences and Environment, University of Kinshasa, Kinshasa, Democratic Republic of Congo; ^6^ Department of Water Resources Engineering, University of Dar es Salaam, P.O. Box 35131, Dar es Salaam, Tanzania; ^7^ Institute for Water Research, Rhodes University, Grahamstown, South Africa

**Keywords:** hydrology, Congo basin, capacity building

## Abstract

In this article, we examine the scientific and sustainable research capacity outcomes of the ‘Congo River: user Hydraulics and Morphology’ or CRuHM project, a six-year effort supported by the Royal Society’s Africa Capacity Building Initiative. This project brought together a consortium of African and UK universities to undertake the first large-scale scientific expeditions to the Congo basin of the modern era in order to better understand the hydraulics and geomorphology of this understudied but globally important river. The river is essential for navigation, irrigation, drinking water and hydroelectric power generation for the 10 basin countries and is critically important for biodiversity and global nutrient, carbon and climatological cycles. This article summarizes the new scientific understanding contributed by the project and the steps taken to ensure a meaningful legacy that would continue long beyond the finite lifetime of available funding. Actions taken to achieve this include establishing a new hydrology research centre at the University of Kinshasa as well as steps to build a wider international community of Congo basin researchers. In this way, we hope to build momentum for future funding initiatives and collaboration.

## 1. An introduction to the Congo river: user hydraulics and morphology project

The Congo is the world’s second largest river by basin area and one of the least studied globally in terms of its hydrology and hydrodynamics [1]. Nevertheless, within the basin, large populations are critically dependent on the river for navigation, power generation, irrigation and drinking water [2] or are at risk from river flooding [3]. In basin countries with limited road infrastructure, the rivers of the Congo system provide the main transport arteries and it has been estimated that the basin contains one-quarter of the world’s hydropower potential [4]. The ecosystems of the basin are also globally significant for biodiversity [5], carbon sequestration [6], nutrient cycling [7] and the planet’s climate [8] and water cycles [9]. The Congo is the world’s second largest lowland tropical forest after the Amazon and the basin has been estimated to store about 80 Gigatons of carbon, an amount equivalent to just over 2 years of total anthropogenic emissions at current rates [10]. All these biological, physical and socio-economic processes are intimately connected to the basin’s water cycle, which itself is being altered as a result of climate and land use changes [11]. In this context, our lack of understanding of the hydrology, fluid dynamics and morphology of the Congo basin’s rivers is a significant barrier to sustainable development and conservation and to understanding the role of the basin as a nature-based solution in the fight against climate change.

The Congo basin has not always been ‘*terra incognita*’ for scientists. Alsdorf *et al*. [1] have shown how in the twentieth century there was, at times, a relatively dense network of river discharge measurement stations operating across the basin. Indeed, the gauging station at Kinshasa has river stage and discharge data going back to 1902, making it one of the longest streamflow records on the African continent. However, over time, the number of river gauging stations within the basin has declined to the point where only a handful are currently operating, and the Congo could now be regarded as one of the most poorly gauged basins in the world. The contrast here to the Amazon basin is instructive. Prior to 1950, there was better scientific knowledge of the hydrology of the Congo basin than the Amazon; however, from the 1980s to 1990s onwards, investment in multidisciplinary, multinational research programmes in South America, such as the Large-Scale Biosphere-Atmosphere Experiment in Amazonia (LBA) [12], have transformed our understanding of the Amazon system. Through LBA and other funding, the application to the Amazon basin of new satellite remote sensing methods [13], field data collection techniques (e.g. [14]) and the organization of large-scale scientific cruises (e.g. [15]) has continued at pace and resulted in important new insights. For the Congo, however, just a handful of remote sensing and modelling studies have so far been conducted (see [1] for a comprehensive review) and until very recently ‘on the ground’ modern scientific surveys were almost non-existent. For this reason, there have been recent calls for a major international investment in basic climate, hydrology and biodiversity science in the Congo basin, of a similar scale to the LBA programme in Amazonia, in order to fill these gaps in knowledge [16].

To begin to address these challenges, the ‘Congo River: user Hydraulics and Morphology’ or CRuHM project was devised (see https://www.crrebac.org/en_GB/hydraulique-et-morphologie-pour-les-usagers-du-fleuve-congo-cruhm). CRuHM was a six-year effort supported by the Royal Society of London’s Africa Capacity Building Initiative, using funding obtained from the UK Government’s Foreign, Commonwealth & Development Office. The project ran from 2016 to 2022 and consisted of a consortium of African and UK researchers working to understand the large-scale hydraulics and geomorphology of the Congo River. The institutions involved were the University of Kinshasa in the Democratic Republic of Congo (DRC), the University of Dar Es Salaam in Tanzania, Rhodes University in South Africa and the Universities of Leeds and Bristol in the United Kingdom. A brief summary of the project is provided below, but for further details, the reader is referred to [2].

The overall aim of the project was to better understand the water and sediment dynamics of the Congo basin, with each African University focusing on a particular set of activities. The University of Kinshasa focused on basin-scale hydrological modelling which was used to provide the forcing data for large-scale hydrodynamic and sediment transport models. The University of Dar Es Salaam was responsible for sedimentological studies, including field data collection and basin-scale sediment modelling, while Rhodes University developed hydrodynamic models of specific Congo floodplain wetlands to improve their parameterization in the basin hydrology model. As well as addressing specific science questions, a further aim of the project was to build significant and long-lasting capacity for hydrology and hydrodynamic research in sub-Saharan Africa. To this end, the project supported the successful training of four PhD students (all co-supervised by different groups of academics drawn from across the project), three technicians and two project managers and created major research facilities at the participating African institutions including laboratories, field sites and survey equipment pools. In particular, along the Congo River main stem the project undertook the first large-scale scientific cruises of the post-colonial era in order to collect new foundational datasets (e.g. bathymetry, flow and sediment concentration) needed to underpin modelling and satellite imagery analysis. Six multi-day scientific training events were held across Africa and made open to local researchers outside CRuHM, and the project also undertook significant outreach to the river’s end users through three large stakeholder meetings. These meetings were held in Kinshasa in the Democratic Republic of Congo in 2016 and in 2021, and in Kigoma, Tanzania in 2019. In total, these meetings involved hundreds of delegates from government ministries, basin organizations (e.g. CICOS - the Commission Internationale du bassin Congo-Oubangui-Sangha, https://www.cicos.int/), local community groups, international NGOs and the media.

From the start of the CRuHM project, there was a clear focus on developing African hydrology research capacity that would continue over the long term and well beyond the finite lifetime of the CRuHM funding. The existing capacity-building literature places considerable emphasis on evaluating current programmes (e.g. [17]), developing strategies to enhance their effectiveness (e.g. [18]) and looking at the experience of participating organizations (e.g. [19]), but very little research has been conducted that examines the long-term legacy and sustainability of the interventions that are made. Nevertheless, it seems rather obvious that when programmes end, the funding needed to employ staff, maintain facilities and engage in meetings and conferences also runs out. For science projects, this is likely to be particularly impactful because of the high basic cost of research, and the larger the programme the greater the ongoing funding needs when a project inevitably finishes. Over time instruments need maintenance or must be replaced, trained staff will need to leave to find employment elsewhere and between-country collaboration becomes more difficult to undertake as travel budgets run out. In other words, unless significant steps are taken to ensure a sustainable legacy, many well-intentioned capacity-building programmes will have a ‘half-life’ of perhaps just a few years.

Having this issue in mind from the very start of the CRuHM project, this paper discusses the steps taken to ensure a lasting impact and legacy. These actions have included helping develop an international community of Congo researchers, establishing scientific institutions and structures at African Universities to act as a locus for future research, building long-term relationships with stakeholders and creating data repositories to allow other researchers access to the unique datasets developed as a result of the project. Section 2 summarizes scientific highlights from the research undertaken within CRuHM and the progress made in capacity building, while §3 details the actions taken to aid the transition from *capacity building* to *capacity sustainability*. Conclusions are drawn in §4.

## 2. Scientific highlights and success in capacity building

### 2.1. An introduction to the Congo basin

Trigg and Tshimanga [20] suggest that the special standing of the Congo basin as an ecosystem of global significance in the context of climate change not only relates to its size but also to the fact that we are only now beginning to understand its uniqueness. As human pressures on the basin grow, we are in danger of losing this system before we have really begun to understand it. Building this understanding remains a challenge in the Congo, where the absence of investment in research over many decades combined with the complexity of natural processes and the remoteness of the basin have so far been the major barriers to science development. The main achievement of the CRuHM project was to conduct the first modern hydrologic science expeditions within the Congo basin to allow the collection of new field data along the main reaches of the river. Some of these river reaches are very complex, remote and difficult to study, with progress requiring significant technical, time and financial resources.

**Figure 1.**
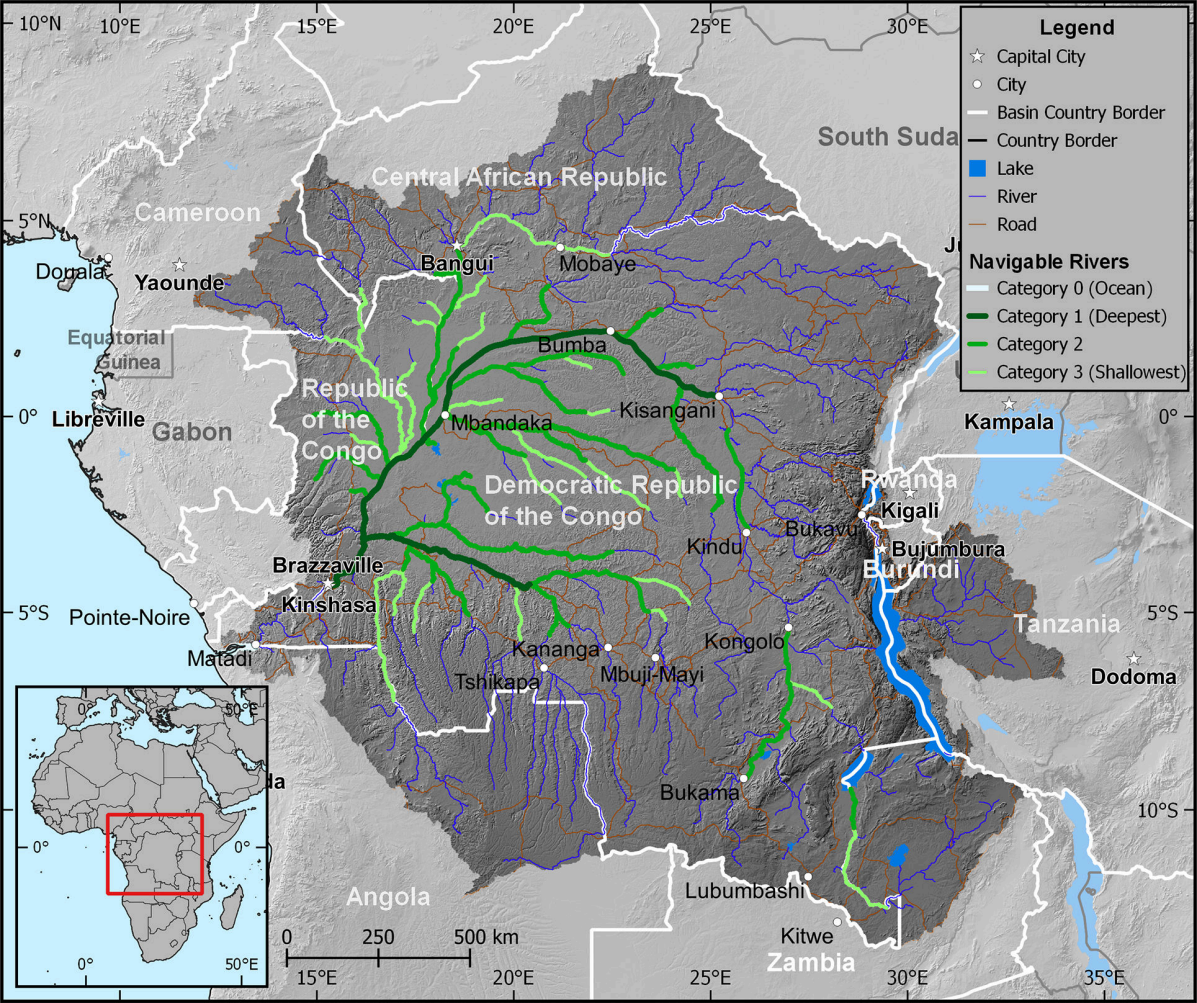
Congo river basin showing major rivers (defined as upstream catchment area >5 000 km^2^), classified navigable river channels and major population centres.

The Congo River can be broadly classified into three main reaches (figure 1). Moving from the headwaters downstream, the first reach begins at the source of the Congo River on the Katanga Plateaus and runs until the Boyoma Falls at Kisangani, at which point the upstream basin area is about 960 000 km^2^. The main stream channel along this first reach is generally known as the Lualaba River and it is only after Kisangani that the river takes the name Congo. The middle reach starts at Boyoma Falls and encompasses the region between the cities of Kisangani and Kinshasa, with a cumulative basin area of about 3.6 M km^2^. Throughout the middle reach, the annual average river discharge increases gradually from 7 640 m^3^/s at Kisangani to 41 000 m^3^/s at Kinshasa, over a river centreline distance of 1 734 km. This increase is caused by the contribution of numerous major tributaries that drain the northern and southern catchments of the Congo basin and which join the main stem along the middle reach. These major tributaries are the Lindji, Awuruwimi, Oubangi, Sangha and Alima Rivers from the north; and the Lomami, Lulonga, Ruki and Kasai Rivers from the south. The river system in the middle reach is characterized by a vast area of large wetlands/floodplains and swampy forests, known as the cuvette centrale, which holds a rich endemic biodiversity and provides diverse ecosystem services. The most extensive peatland complex in the tropics has recently been discovered in the forests of the cuvette centrale and shown to store carbon equivalent to that of the basin’s above-ground rainforest biomass [6].

This middle reach of the Congo River is also a key driver of the regional blue economy in central Africa. In such a humid tropical environment that critically lacks road infrastructure, the middle reach of the Congo River and its tributaries are an essential lifeline that connects several riparian countries and serves as the main navigation corridor for the transport of goods and exchange of services. As a result, it has also been used since the colonial era for international trade. A circular riverine water body, the Malebo Pool, marks the end of the middle section before the Congo River enters its lower reach downstream of Kinshasa.

Uniquely amongst large rivers, along this final stretch of its course, the river steepens and the elevation drops around 270 m over its last ~500 km, passing through a series of major cataracts before reaching the basin outlet into the Atlantic Ocean. As a result of the hydraulic head this generates, the lower reach has significant hydropower capacity installed along it, and in particular, the two hydroelectric plants at Inga Falls have the potential to generate ~39.6 gigawatts of electricity. The river finally enters the Atlantic close to Kitombe in the DRC where the cumulative basin area is 3.7 M km^2.^

### 2.2. Science highlights

Fieldwork campaigns for the CRuHM project were carried out along the middle reach of the Congo River, between Kisangani (upstream) and Kinshasa (downstream). Fieldwork also targeted measurements in the Kasai, a first-order tributary of the Congo, where an automatic sediment sampler was installed. This was used for continuous monitoring of flow and sediment in a part of the basin where water pollution due to mining activities constitutes a major challenge [21]. For example, in July and August 2021, this region of the Kasai River experienced an environmental disaster due to water pollution with high turbidity and heavy metals caused by the Catoca mine tailings spill, which led to a loss of life, aquatic biodiversity and huge impacts on the livelihood of riverine communities. The Catoca spill became an issue of transboundary water conflict between Angola and DRC, and the CRuHM sediment data were used to provide evidence of the pollution [22].

The fieldwork conducted during the CRuHM project has enabled the development of an unprecedented database of discharge measurements, including river channel cross-sectional velocity profiles using Acoustic Doppler Current Profiler (ADCP) technology; water level logger data: continuous water surface elevation measurements over time at specific locations; samples of the bed and suspended sediment loads; water surface elevations from Global Navigation Satellite System (GNSS) receivers at regular intervals along the Congo; river channel bathymetry; and river bank cross sectional topography. We conducted numerous science cruises with large teams of researchers (>20 scientists) drawn from the project participating institutions and government organizations within the DRC. In particular, our fieldwork benefited greatly from the contribution of river navigation authority in the DRC, the Régie des Voies Fluviale.

Highlights of the science conducted based on these new data include a characterization of the Congo basin catchment units necessary to understand the interaction between climate variability, catchment properties and the resulting hydrological response [23]. Given the largely ungauged nature of the Congo basin, this understanding was used to map hydrological characteristics across the basin and establish their likely uncertainty ranges. In turn, this was used to constrain computer models of the basin’s water cycle to simulate, quantify and ultimately reduce uncertainty in hydrological predictions [24,25].

**Figure 2.**
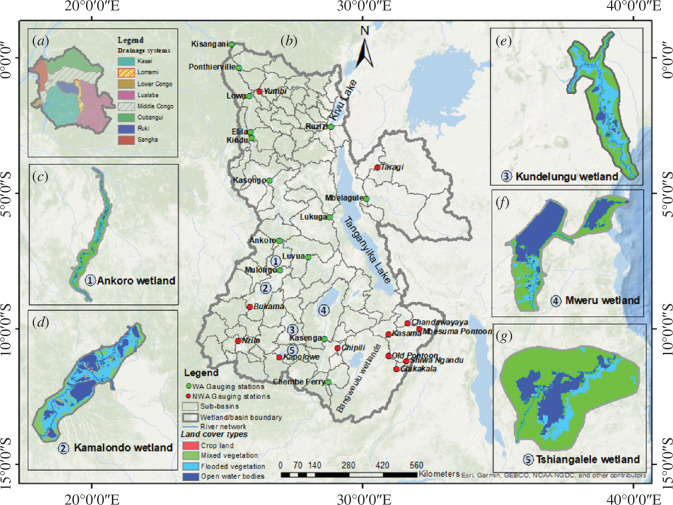
Location of the studied wetland systems in the Lualaba drainage system. WA and NWA stand for wetland affected and non-wetland affected gauging stations, respectively. (*a*) Location of the Lualaba drainage system within the Congo Basin. (*b*) Lualaba drainage system with main river flowing from south to north with Kisangani gauging station as the outlet. (*c*)–(*g*) showing the extent of the studied wetland systems.

**Figure 3.**
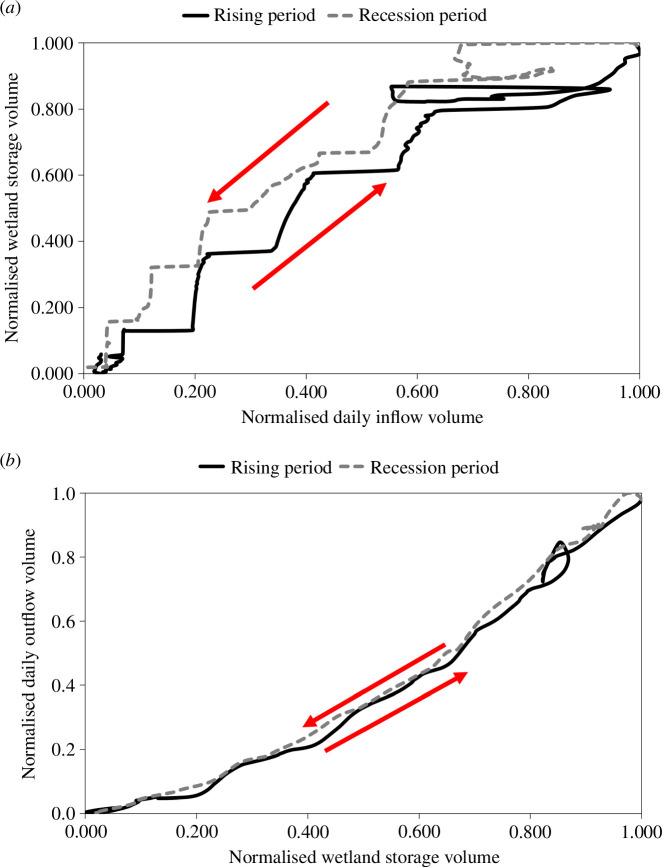
Anticlockwise Hysteresis representing inundation (*a*) and storage release (*b*) processes of the Kundelungu/Lufira wetland system.

A further contribution to scientific understanding that came out of the CRuHM project was the inclusion, for the first time, of channel-wetland exchange processes within a basin-scale integrated hydrological modelling framework. These wetlands (namely, the Ankoro, Kamalondo, Kundelungu/Lufira, Mweru and Tshiangalele wetlands located in the Lualaba drainage system in the Congo upper reach, see figure 2) are known to play a critical role in flood wave generation within the Congo river basin through their ability to store transient water and modify downstream flow regimes. This scientific understanding can be expressed in terms of hysteresis (figure 3), which characterizes the ingress, storage and release of water from the wetlands [26]. The size of the hysteresis loop represents the wetland’s ability to store and release water which then attenuates flood wave propagation and reduces downstream inundation. This effect is scale-dependent, with minor wetlands only developing small hysteresis loops which have a small attenuation effect and result in fast release of water to downstream reaches.

**Figure 4.**
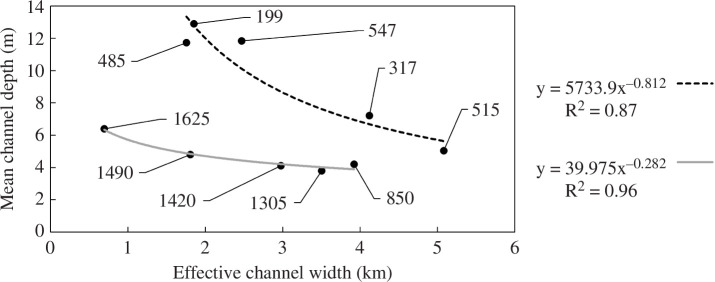
Observations of cross-sectional channel width and mean depth at 10 locations along the middle Congo, with two power law relationships fitted. The two relationships shown cover the upper and lower halves of the middle reach, and the change in relationship around the city of Mbandaka is attributed to the large tributary inflows in this area (namely from the Oubangui, Lulonga and Ruki rivers). Each observation is labelled with its distance from Kinshasa in kilometres, and each relationship is labelled with its equation and coefficient of determination (R^2^).

In addition, the field data have been used to characterize hydraulics, assess satellite altimetry datasets, provide bathymetry for hydrodynamic models and model fluvial hydraulics and hydrodynamics, all of which are necessary to understand biogeochemical cycling, ecology, public health, transportation and flooding within the basin [27,28]. Our hydraulic characterization and hydraulic modelling work on the middle Congo mainstem led to several significant findings. We discovered that four prominent contractions in channel width located between the Oubangui and Kasai confluences do not form hydraulic control points that constrict flow and are not a cause of fluvial floodplain inundation in the Cuvette Centrale as previously thought [29]. We discovered that strong hydraulic geometry relationships between effective channel width and mean depth exist along the middle Congo mainstem as shown in figure 4. The two relationships shown cover the upper and lower halves of the middle reach, and the change in the relationship around the city of Mbandaka is attributed to the large tributary inflows in this area (namely from the Oubangui, Lulonga and Ruki rivers). Documentation of such relationships is rare in large river multi-threaded channel environments such as those that dominate the middle Congo [30–32]. This provides a valuable insight into the validity of hydraulic geometry relationships in these anabranching systems, which are surprisingly common along the world’s largest rivers [33]. The relationships also have broad practical utility as a means of obtaining an estimate of channel depth from more readily available width observations. Depth information is critical to water resources and risk management activities such as discharge monitoring and flood inundation prediction yet is very difficult to impossible to observe at scale [34,35]. By contrast, river width is widely observable from space with satellites [36–39].

We also found that the longitudinal water surface profile of the middle Congo mainstem is well described by water surface elevation datasets from satellite profiling altimeters (within 0.3 m of *in situ* observations along 95% of the reach); although the planform transition from multichannel to single-channel upstream of the Kasai confluence is an important exception to this, with significant variations in water surface slope observed here in ground surveys.

**Figure 5.**
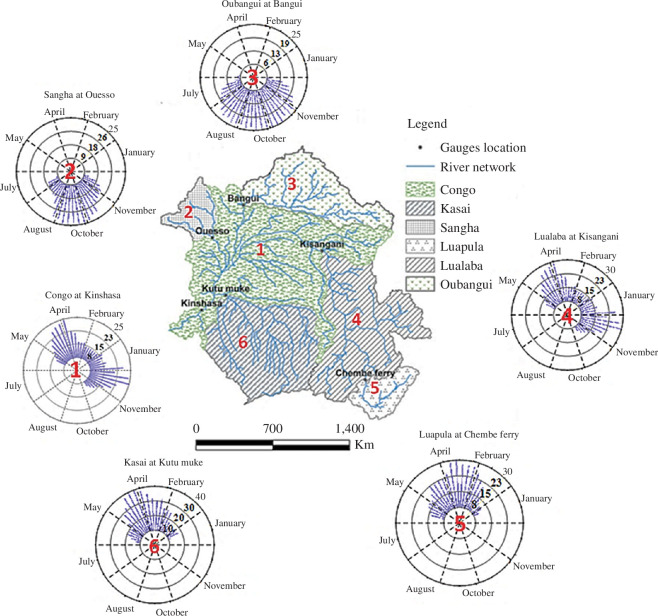
Spatial patterns of flood hydrograph shape across the basin documenting the switch from a monomodal to a bimodal flood. Values are expressed in relative frequency by date (%).

The Congo is unique among large rivers because of its almost circular course, which crosses the equator twice, and a feature of the system that is a significant contributor to the formation of the river’s double-peaked annual flood pulse. Our measurements and modelling of the Congo’s catchment hydrology and hydraulics have helped uncover the mechanisms behind the double annual flood pulse and have enabled the first characterization of flood patterns across the basin to determine where in space the switch from a monomodal to a bimodal flood pulse occurs (figure 5, 40,41) . In contrast to the bimodal flood pulse seen along the Congo main stem, our work showed that only a unimodal flood pulse is produced by the Northern and Southern tributaries contributing flow along this reach. We, therefore, conclude that the bimodal flood pulse is generated by the flood waves from the contributing catchments arriving at the main stem at different times and combining either synchronously or asynchronously with the main stem flow peak.

**Figure 6.**
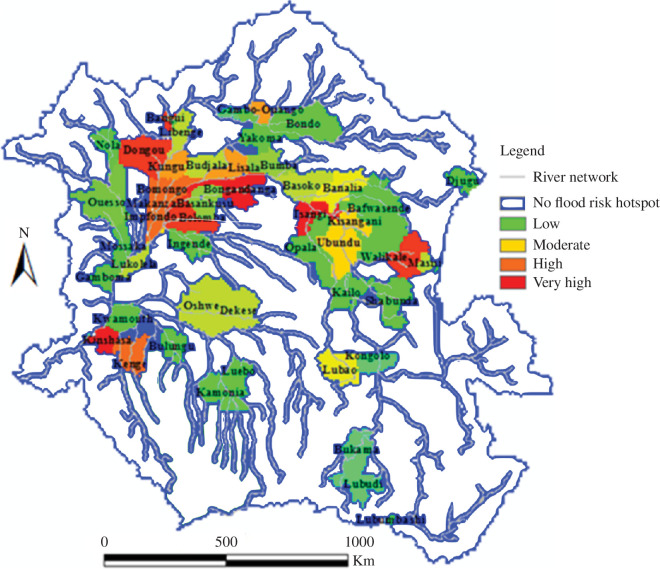
Hotspots for 100-year flood risk in the Congo river basin.

Given that 100 million people in the Congo basin rely on Congo River, information on flood patterns supports numerous river-based activities and services. Timing of floods has an effect on floodplain farming systems as well as the livelihoods of inhabitants who modify their agricultural and floodplain activities to correspond with the rise and fall of the flood wave [42]. The flood pulse can affect biotic composition, nutrient transport, fish production, animal habitat creation, floodplain construction and soil fertility restoration. Flood risk planning and preparedness are required not just in ‘hotspot areas’ but also throughout ‘hot seasons’. Our measurements and modelling of the Congo’s catchment hydrology and hydraulics have therefore also helped to predict flood risk [3]. Flood risk analysis has led to the creation of hotspot maps (figure 6) by considering areas in which high occurrence of flooding coincides with high exposure based on flood hazards and risk analysis.

**Figure 7.**
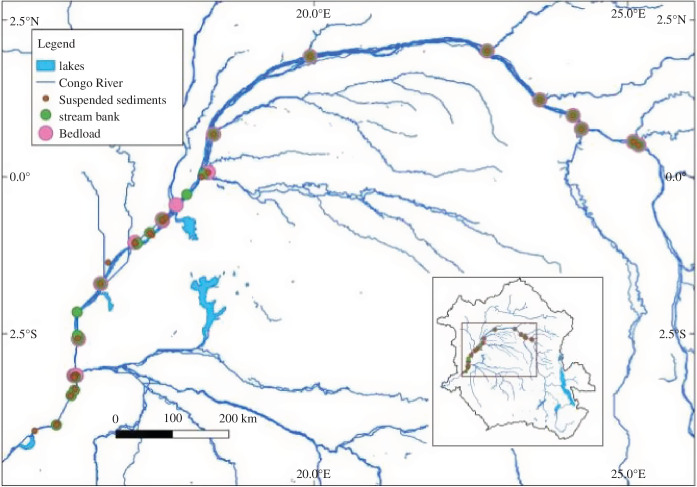
Sediment sampling locations on the Congo mainstem.

Lastly, through field sampling, we have been able to characterize the Congo’s sediment distribution and how this could impact future hydropower development [21,43]. The study used a conceptual framework developed in the Pangani river basin in Tanzania for sediment studies in poorly gauged catchments like the Congo basin. The framework consisted of a conceptual model, a network of sediment property and yield flux monitoring sites across the basin and a feedback loop to connect the two components. The sampling programme (see figure 7) consisted of two parts. First, measurements were taken near the outlets of major sub-basins to validate the sediment load contribution of each tributary to the Congo mainstem and, second, a detailed sedimentological study was conducted in the Kasai sub-basin.

The study resulted in the first mapping of sediment sources and erosion process within the Congo basin, with time-lapse satellite images revealing spatial and temporal increases in water turbidity of the main tributaries of the Congo river over the past 35 years [43]. The study also identified that the main sediment sources in the basin are in the upper parts and that sheet erosion was the main erosion mechanism, although gully erosion of strong to extreme severity also occurs to a lesser extent [21]. The Kasai and the Upper Congo sub-basins are the largest contributors of sediment load into the Congo mainstem. Current sedimentation rates in the basin may not pose a significant danger for planned and existing hydropower projects downstream in the medium- to long-term but this is dependent on the management of anthropogenic influences, particularly from mining and deforestation.

A final question addressed by the CRuHM project is how the Congo basin will respond to development and climate pressures in the region. Debates in this area point to trade-offs between conservation of the basin’s natural environment—ecosystem intactness—and improving the livelihood of communities in the basin through optimal use of its resources. Answering this question requires data to be available not only on the physical characteristics of the Congo basin but also on the dynamics of land use and socio-economic settings. As of today, through land use and population data collected and analysed under the CRuHM project, we now know that the Congo basin holds approximately 120 million inhabitants, of whom about 83% live within a distance of 50 km of a major tributary in the basin and 33% live within 10 km of a navigable waterway [2]. At the same time, these tributaries are subject to morphological changes brought about by land use and climate change. Trigg *et al*. [44] used morphometric analysis of river planform change to compare the river geomorphology depicted in century-old navigation charts with channel locations in modern remote sensing images and showed high levels of sediment deposition, particularly within wide shallow river reaches that are already difficult to navigate. The major challenge here is the exposure of human population and biodiversity to various climate- and water-related risks, impacts of change on the fragile economy of the basin’s countries, and the type of prediction and planning tools to be developed to prevent losses and damage. For instance, a single flood event in May 2023 in the Democratic Republic of Congo caused over 400 deaths and a significant amount of economic damage. Indeed, these risks are complex and extensive and need to be accurately addressed. The new data collected and knowledge generated through the successful implementation of collaborative research and capacity building within the CRuHM project therefore represents a major advance in science development for the Congo basin. At the same time, this information also provides an improved understanding of the real scale of challenge that science needs to address for equitable development in the basin. The work carried out under CRuHM is just a beginning towards what is required to address these challenges and fill science gaps for this vast and remote hydrological system.

### 2.3. Capacity building

Under the CRuHM project, the capacity-building programme addressed the training of individual researchers, laboratory technicians and professionals from the collaborating institutions; strengthening research institutions to sustain scientific research and innovation in the basin and undertaking knowledge dissemination. In total, four PhDs (three men and one woman) were trained under the CRuHM project. From the start, the selection of the PhD students was made such that they would come from the partner universities and contribute to strengthening the capacity of their own institutions after completion of their studies. This is critical as the field of hydrology critically lacks skilled human capacity in Sub-Saharan Africa. Three PhD students were funded directly by the project, and an additional aligned PhD student was funded by a University of Leeds scholarship.

In addition to typical PhD training, the project organized several short courses and training workshops aimed at enhancing the skills of researchers on data collection for large rivers, analysis and modelling and process understanding. Some of these training activities were organized by the partner universities in South Africa, Tanzania, the DRC and the United Kingdom, with each university addressing a specific topic of the project implementation. Other training sessions were held in the field during our scientific expeditions. It should be mentioned that the CRuHM project provided a great opportunity for Sub-Saharan researchers to use state-of-the-art equipment for investigation of large rivers, which required specialist training. Such training involved the use of Acoustic Doppler Current Profilers (ADCPs) for discharge measurement, sonar systems for bathymetric mapping and GNSS technologies for geolocation and surveying, the installation of automatic water level loggers, the use of sediment pumps and installation of an automatic integrated sediment sampler (known as an ISCO) for continuous sediment monitoring. Currently, our ISCO station remains the only water-monitoring facility that is operational in the Kasai River, collecting turbidity, sediment and flow data at an hourly time step and providing large-scale monitoring for a river that drains a basin area of over 900 000 km^2^.

The second dimension of the CRuHM project capacity-building strategy targeted strengthening research institutions to sustain scientific research and innovation in the basin. The main achievements in this regard were the establishment of the Congo Basin Water Resources Research Center (CRREBaC, www.crrebac.org) at the University of Kinshasa and the NTWAM Water and Environment Initiative in Tanzania (https://ntwam-water-env.or.tz/), both of which serve to ensure the legacy of research carried out under CRuHM. Prior to the CRuHM project, there was a complete lack of an organizational entity to conduct scientific research into basin-wide water resources issues. CRREBaC was established in 2018 following the successful mid-term review of the CRuHM project where the need to establish a legacy for research and innovation in the basin was identified, while NTWAM was founded in October 2023 to serve as a platform for water and environment research, knowledge exchange and evidence-based water policies and practices in Tanzania.

The overall mission of CRREBaC is to contribute to the sustainable management and development of water resources in the Congo basin through research that provides scientifically robust information and innovative solutions to emerging water resource problems. In recent years, CRREBaC has successfully engaged in research collaboration with various international and regional partners that have contributed to the implementation of a number of further research and capacity-building projects in the Congo basin. CRREBaC was designed to act as a gateway for research in the Congo basin, bringing together international and local researchers with an interest in advancing science in the basin and supporting them with available historical data and other *a priori* information. For example, the work done by CRREBaC during the last three years persuaded the DRC government, through its Ministry of Higher Education, to fund a capacity-building programme to train a critical mass of professionals and scientists. This has resulted in the creation of the Regional School of Water (ERE, Ecole Régionale de l’Eau, https://crrebac.org/ere) at the University of Kinshasa. ERE, therefore, constitutes an important tool for the implementation of policies for sustainable water resources management and improved access to water services in the DRC and the Congo Basin more generally. The ERE’s mission is to develop human capital specialized in water resources management and water engineering, to equip them with modern tools and enable them to respond to current and future challenges, to understand and manage water resources in an integrated and cross-cutting manner, with a view to maximizing the resulting economic and social well-being without compromising the sustainability of vital ecosystems. The ERE programme focuses on training and research for Masters of Science and PhDs in Water Resources and a Professional Masters programme in water governance. It also offers training in the field of water with a view to meeting emerging needs.

For the most recently completed academic year (2022–2023), ERE had registered 45 Masters’ students coming from a broad range of water resources and environmental disciplines, including 12 agricultural engineers, 6 civil and hydraulic engineers (polytechnics), 5 oil and gas engineers, 3 geologists, 2 hydrologists, 4 building and public works engineers, 8 environmental engineers, 1 geographer, 2 chemists and 2 economists. These students originate mostly from the DRC but also other African countries under the collaborative framework of the African Water Resources Academic Mobility Network (AWARMN, https://www.ru.ac.za/intra-africa-awarmn/) which links partner institutions including Rhodes University in South Africa, Makerere University in Uganda, University of Kinshasa in the DRC, the Federal University of Technology in Nigeria, the National School of Hydraulics in Algeria and TU Delft in the Netherland.

Lastly, the capacity-building strategy under the CRuHM project targeted knowledge dissemination and awareness raising of the importance of advancing science to achieve sustainable development in the Congo Basin. Through this pillar, we held scientific conferences, media campaigns, published in newspapers to reach out to non-scientists and contributed to a major book that lays down the foundation of hydrological science for the Congo basin [45]. These materials are now widely used by scientists, investors and international organizations to guide future endeavours in the basin.

## 3. From capacity building to capacity sustainability

The international and national context of a scientific consortium collaboration project such as CRuHM is perhaps just as important as the internal management and operation of the project and has long-term implications for the sustainability of these endeavours. It is, therefore, worth touching on this context as part of our reflections on the successes, challenges and long-term legacy of the project. Much has been written on North–South (N–S) research collaborations over the last decades, much of this in the health sector [46–48]. Indeed, the consensus on how these partnerships should be approached and conducted has evolved significantly over time in response to experience and reflection, as well as a changing international influence in development [49].

Scientific collaborations between North–South institutions are not a new concept and these have evolved from colonial times, commonly through development aid. However, these efforts have often been criticized for being one-sided and neocolonial in nature with an asymmetric relationship in terms of power and resources [47,48]. This was recognized relatively early and enshrined, for example, in the Vienna Program of Action, which urged that N–S cooperative research should have four key characteristics; it should (i) be in keeping with development priorities determined by developing countries, (ii) provide for developing country participation, (iii) provide for joint participation and control, and (iv) include a training component. It has taken a long time for this to evolve into the current practice and there are still many remaining issues that can derail a project such as CRuHM. While the situation today is somewhat better than in the past, often with funders requiring explicit attention to these issues in the programme design and implementation, there are still many challenges that impact the long-term sustainability of the science and staff involved in these joint programmes. We will touch on a few relevant examples from our experience and how these affected the project and describe the actions we took to address these.

We constructed the CRuHM programme through a co-development process through discussion of mutual overlapping interests, which took time. This was not a contrived shoehorning of ideas into a joint project, but a genuine dialogue developed over two years. The funding call had two rounds over two years, and we initially prepared to submit for the first round but missed the deadline due to a sudden illness of one of the partners. This did, however, allow us more time to develop a plan that was better aligned to the African partners’ needs and complementary expertise. We have had some success applying for other funding towards the end of the project but noticeably call announcements and submission deadlines seem to get ever shorter. This inevitably means less time to develop a project that is truly equitable as it pushes for the PI to complete as much as possible for the rapidly approaching deadline, and it can be tempting to short circuit the consultation process as a result. With existing partnerships this is perhaps less of a problem as there is a history of working together, but when trying to develop new partnerships this can mean collaborations start off with a built-in bias. We recommend that funders consider this challenge when developing their funding calls and allow time to develop truly balanced partnerships.

As is commonly the case with these projects, funds were channelled through the UK partners, ostensibly for practical administrative reasons. However, as well as adding additional administrative burden to the UK institutions, this inevitably reinforces the imbalance of power discussed above. We were able to ameliorate this to some extent by ensuring equal division of funds between partners, joint financial decision-making and transparency with budgets. For example, this enabled the consortium to fund a fieldwork vehicle that was not originally envisaged at the start of the project. This was achieved through a mutually agreed redistribution of funds from all partners and was only possible with the open trust and transparency that had been developed and with support from the funder.

The project also faced some funding and logistical challenges. For example, some of our follow-on project funds were affected by the UK government’s realignment of priorities towards overseas aid. These budget cuts, while being problematic for the UK participants, were devastating to our African partners and certainly damaged relationships and trust in the funding process. An Ebola outbreak in Mbandaka in the DRC in 2018 and COVID-19 from 2020 onwards also affected our fieldwork and meeting programme. Planned fieldwork in 2018 focused on the stretch of river around Mbandaka and had to be moved, and all meetings in the final two years of the project had to be moved online as international travel was not possible. In particular, our final project meeting had to shift from an in-person event in Kinshasa to a more complex and somewhat limited hybrid online/in-person meeting with simultaneous translation of French and English speakers.

Science in a development context, especially when aligned with African partners’ needs, is often seen as applied science that is somehow not equal to pure ‘blue skies’ research, and there remains a tension between these two camps. Without getting into the technicalities of this argument here, we would argue from our experience that it is possible to achieve both types of research simultaneously, as demonstrated in the previous section. We believe work in CRuHM shows that one can produce important journal papers with original pure science findings, while also addressing key development issues like navigation, hydropower and water resources. The concept of good quality science for the population of the river system has been at the core of our project from the beginning and indeed the consortium was put together to bring specific but complementary science expertise contributed uniquely from each of the partners. As noted by Keay of the Royal Society, as far back as 1965 [50] but unfortunately still applicable today (see Alsdorf *et al*. [1]), ‘With much of the basic fabric of science in Africa inadequately known, and yet different from the better known regions of the world, there is always a possibility that imaginatively pursued applied research may lead on to fundamental discoveries of major importance*.*’

Getting the balance right between pure and applied science required a continuing effort throughout the project. For example, to ensure relevance, we developed strong long-term relationships with stakeholders, included stakeholders in research activities, shared data, conducted workshops and provided capacity building for a wider group beyond those funded by the project. We also encouraged CRuHM PhD students to aim for high-quality journal outputs (with project budget ring-fenced for open-access charges). This sometimes brought to the fore issues with predatory journals and required extra supervision and guidance effort, with the goal of giving the project PhD students a strong start to their academic careers.

In terms of next steps after the project for our PhD students, we again see a contrast between North and South, with the UK project-aligned PhD moving to a classical postdoctoral role in another project, but with the African PhD students moving (or even returning) to a teaching heavy role within their universities, with less opportunities to develop the science research aspects of their career. This is somewhat outside of our direct control in the project and is more a result of the academic context, but nonetheless important. Another aspect outside of our control, but relevant for our joint fieldwork campaigns, was the unequal availability of fieldwork insurance (despite the funds available); this in reality meant that our African partners were taking much bigger risks than personnel from the United Kingdom. We deliberately included our PhDs and technicians in planning meetings to expose them to the realities of research projects and indeed this was an integral part of our transparency process, not just between partners but also within partner teams. In applying for follow-on funding, we have also actively included the PhD students in this process, for example, as named postdoctoral positions, although there is the added challenge for them of taking a temporary research position over a permanent and more stable teaching position.

Lastly, to further develop hydrologic science capacity in sub-saharan Africa and create a sustainable legacy for the CRuHM project, we have attempted, along with colleagues around the world, to increase the international visibility of Congo basin science and create a community of researchers studying this important and under-explored region. These actions have had the aim of building momentum for larger funding and cooperation initiatives that could better sustain research at our African partner (and other) institutions into the future. Specific tasks have included the co-organization of a major international conference on Congo hydrologic research held in Washington, DC in September 2018 under the auspices of the American Geophysical Union. This led directly to the publication of an extensive monograph covering many aspects of the Congo’s hydrology, climate and biogeochemistry [45] which has led to increased research interest in the basin. In addition, we have created an open web portal that hosts much of the data generated by the CRuHM project so this is available to other researchers. This site, known as the Congo Basin Catchment Information System (CB-CIS, https://cbcis.info/), provides comprehensive information on the water resources of the basin for scientists, decision-makers and communities. Lastly and again with colleagues from around the world, CRuHM project members have contributed significantly to the development of two new sister initiatives: (i) the Congo Basin Science Initiative (https://congobasinscience.net/), inspired by the successful Large Scale Biosphere Atmosphere project in Amazon which aims to stimulate investment to understand the Congo Basin as a regional entity and train a new generation of scientists from the region; and (ii) the Science Panel for the Congo Basin, inspired by Science Panel for the Amazon and convened by the United Nations Sustainable Development Solutions Network, which aims to synthesize existing knowledge of the Congo basin and its ecosystems with the goal of producing an independent scientific assessment report to be presented at COP30 (https://www.spcongobasin.org/).

## 4. Conclusions

Capacity-building programmes within the sciences face significant challenges when funding inevitably comes to an end, and the larger the programme the greater the future funding gap. Within the ‘Congo River: user Hydraulics and Morphology’ project funded by the Royal Society’s Africa Capacity Building Initiative, steps have been taken to anticipate and mitigate this challenge in order to create a sustainable legacy. These actions have included:

— Developing extensive links with stakeholders within the basin, including with the DRC River Navigation Authority (the Régie des Voies Fluviale), CICOS (the International Commission for the Congo-Oubangui-Sangha Basin) and government ministries in the DRC and Tanzania in order to improve professional training and knowledge.
— Development of open data portals to host the information collected by the CRuHM project.
— Efforts to build an international community of Congo basin researchers and momentum for large-scale funding and cooperation.
— Establishing a new research centre for Congo basin hydrologic science (the Congo Basin Water Resources Research Center or CRREBaC, see www.crrebac.org) at the University of Kinshasa in the DRC to contribute scientific evidence for the sustainable management and development of water resources in the Congo basin.

Given the project ended only in 2022, it is still too early to judge the success of these actions, although initial signs are promising. However, despite the challenges discussed and a continuing unequal science context, we believe CRuHM was able to build a strong and equal North–South partnership, based on friendship and trust, and we often refer to our project team as the CRuHM family. We continue to work together, sometimes with explicit research funding—but also without, determined to support each other and our research, building on the project legacy through initiatives such as CRREBaC and other relevant activities.

## Data Availability

This article has no additional data.
